# Colorectal Cancer Screening Among People With Intellectual Disabilities

**DOI:** 10.1001/jamanetworkopen.2025.57013

**Published:** 2026-01-30

**Authors:** Trine Allerslev Horsbøl, Susan Ishøy Michelsen, Trine Toft Sørensen, Knud Juel, Morten Rasmussen, Ismail Gögenur, Susanne Oksbjerg Dalton, Amina Banda, Maarten Cuypers, Lau Caspar Thygesen

**Affiliations:** 1 National Institute of Public Health, University of Southern Denmark, Copenhagen, Denmark; 2 Department of Clinical Medicine, University of Copenhagen, Copenhagen, Denmark; 3 Digestive Disease Center, Bispebjerg University Hospital, Copenhagen, Denmark; 4 Center for Surgical Science, Department of Surgery, Zealand University Hospital, Køge, Denmark; 5 Cancer Survivorship, Danish Cancer Institute, Copenhagen, Denmark; 6 Department of Clinical Oncology and Palliative Care, Zealand University Hospital, Næstved, Denmark; 7 Department of Primary and Community Care, Radboud University Medical Center, Nijmegen, the Netherlands; 8 Academic Collaborative Intellectual Disability and Health–Sterker op eigen benen, Nijmegen, the Netherlands

## Abstract

**Importance:**

Colorectal cancer mortality is elevated among people with intellectual disabilities, potentially because of delayed diagnosis.

**Objective:**

To compare colorectal cancer screening participation and completion among people with and without intellectual disabilities.

**Design, Setting, and Participants:**

This nationwide, register-based cohort study was conducted in Denmark, where all residents aged 50 to 74 years are invited to biennial, free-of-charge colorectal cancer screening. People with and without intellectual disabilities born between 1940 and 1973 and invited to colorectal cancer screening at least once between 2014 and 2023 were included.

**Exposure:**

Intellectual disability, defined as being registered with an intellectual disability diagnosis or a diagnosis most likely leading to intellectual disability. Disability severity (mild, moderate, severe, and profound) was available for a subpopulation.

**Main Outcomes and Measures:**

The primary outcomes were colorectal cancer screening participation and completion, including stool sample return, screening results, and diagnostic examination (mainly colonoscopy) following a positive screening test result. The pseudo-observations method was used to estimate cumulative incidence differences and cumulative incidence ratios between people with and without intellectual disability.

**Results:**

The study included 17 117 people with (median [IQR] age, 55.2 [50.2 to 63.2] years; 8445 female [49.3%]) and 149 162 without (median [IQR] age, 54.8 [50.2 to 62.5] years; 75 324 female [50.5%]) intellectual disabilities. Among those, 5170 people with intellectual disabilities (30.2%) and 83 709 people without (56.1%) returned a stool sample within 90 days of first invitation (adjusted cumulative incidence difference, −23.2 percentage points; 95% CI, −24.0 to −22.4 percentage points), with participation increasing with disability severity (range, 1453 of 5293 individuals with mild [27.5%] to 198 of 488 individuals with profound [40.6%] intellectual disabilities). Nonanalyzable samples were more common among people with vs without intellectual disabilities (105 individuals [1.8%] vs 330 individuals [0.4%]). Among those with a positive screening test result, 347 people (70.5%) with intellectual disabilities and 4724 (90.2%) without underwent diagnostic examination (mainly colonoscopy) within 60 days (adjusted cumulative incidence difference, −17.9 percentage points; 95% CI, −22.1 to −13.7 percentage points). The proportion who underwent diagnostic examination decreased with increasing disability severity (range, 127 of 165 individuals with mild [77.0%] to 50 of 100 individuals with moderate to profound [50.0%] intellectual disabilities). Colonoscopies were more often incomplete among people with vs those without intellectual disabilities (109 individuals [28.2%] vs 673 individuals [13.8%]).

**Conclusions and Relevance:**

In this cohort study of people with and without intellectual disabilities, those with intellectual disabilities were less likely to participate in colorectal cancer screening and, when they did participate, more often encountered challenges with stool sample collection and colonoscopy completion. These disparities call for tailored, decision-supportive strategies to ensure equitable access to colorectal cancer screening.

## Key Points

**Question:**

Do people with intellectual disabilities participate in and complete colorectal cancer screening to the same extent as those without such disabilities?

**Findings:**

In this register-based cohort study of 17 117 people with intellectual disabilities, screening participation and completion were significantly lower compared with a reference cohort. The proportion returning a stool sample increased with intellectual disability severity, whereas the proportion undergoing diagnostic examination after a positive screening test result decreased.

**Meaning:**

These findings indicate substantial inequities, highlighting the need for tailored strategies to ensure equitable colorectal cancer screening access for people with intellectual disability.

## Teaser

This cohort study examines participation in and completion of colorectal cancer screening among people with intellectual disabilities in Denmark.

## Introduction

Globally, at least 1% of the population have intellectual disabilities (ID).^1,2^ Causes of ID are primarily prenatal and perinatal, and the condition is characterized by limitations in intellectual and functional ability.^3^ Colorectal cancer (CRC)–related mortality is twice as high in this group than in the general population.^2,4^ This could be explained by an elevated CRC incidence among people with ID, as the prevalence of risk factors such as obesity^5^ and physical inactivity^6^ are increased in this group. However, findings regarding CRC incidence in this group are based on small sample sizes and are inconsistent.^7,8,9^ Another contributing factor could be less-efficient cancer diagnostics in this group leading to detection of cancer in late stages, limited treatment options, and poor survival. A recent Canadian cross-sectional study of patients with CRC showed that patients with ID were 1.44 times (95% CI, 1.24-1.67) more likely than those without ID to receive a diagnosis at stage IV.^10^

CRC screening has demonstrated efficiency to reduce CRC incidence by enabling identification and removal of precancerous lesions, and to enable early detection and reduce CRC mortality.^11^ Despite universal access to the Danish national CRC screening program, some groups in the society are less likely to participate, including people with mental disorders, immigrants from non-Western countries, people without a partner, and those with the lowest income, or with less education.^12,13^

Previous studies on ID and CRC screening are from Canada, US, Europe and Australia, and they consistently report lower screening participation among people with ID (14%-52%) compared with those without ID (27%-73%).^14,15,16,17,18,19,20,21,22^ However, results on CRC screening participation across subgroups of people with varying levels of ID remains limited. Moreover, existing research has primarily concentrated on the initial step of the screening pathway, without addressing subsequent steps following a positive screening test result.

People with ID are a vulnerable group in the health care system.^23^ They have special needs, and to various extents they are dependent on support in activities of living. Currently, no tailored strategies for cancer prevention and screening exist for people with ID. Knowledge that can inform such initiatives is urgently needed.^24^ The objective of this study was to investigate screening participation and completion within the Danish CRC screening program, comparing people with varying levels of ID severity with those without ID.

## Methods

We conducted a nationwide register-based cohort study among people with and without ID. Information from the registers was merged on an individual level through a unique personal identification number assigned to all Danish residents at birth or immigration.^25^

According to Danish law, ethical review and informed consent from participants are not required in register-based studies. In agreement with the General Data Protection Regulation, the present study is registered at the University of Southern Denmark. The study follows the Strengthening the Reporting of Observational Studies in Epidemiology (STROBE) reporting guidelines for cohort studies.

### Screening Program

The Danish CRC screening program is tax funded and offered free of charge biennially to all Danish residents aged 50 to 74 years. It was implemented from March 3, 2014, over a 4-year period, and has been active since then. The target population is invited by mail; however, it is possible to opt out of the program to avoid future invitations. The procedure consists of a home-based stool sample. Participants return the sample by mail, and it is analyzed using a fecal immunochemical test (FIT) to detect invisible amounts of blood. The threshold for defining a positive screening test is 20 µg of fecal hemoglobin per gram of feces. People with a positive FIT are invited to a colonoscopy by mail within 14 days.^26^

### Population

The source populations for this study are 2 existing cohorts: a nationwide cohort of people with ID, and a reference cohort of 10:1 sex-matched and age-matched people without ID. The ID cohort members were identified from a range of registers.^2^ The majority were identified in the Danish National Patient Register^27^ or the Danish Psychiatric Central Research Register,^28^ where they were registered with ID diagnoses or a diagnosis most likely leading to ID (eTable 1 in Supplement 1). Furthermore, people who were granted disability pension because of ID were included from the Danish Agency for Labor Market and Recruitment, and people with cerebral palsy and ID were identified in the Danish Cerebral Palsy Registry. Finally, people living at institutions for people with ID were also included. Information on ID severity was available for people registered with *International Statistical Classification of Diseases and Related Health Problems, Tenth Revision (ICD-10)* codes F70 to F73 (mild, moderate, severe, and profound ID). The reference cohort was sampled from the Danish Civil Registration System^29^ among people who were alive and living in Denmark from January 1, 1976, onward.

From these 2 source populations, we included people born between 1940 and 1973, who were alive when they entered the target population for CRC screening (age 50 years or March 3, 2014), and who were invited to CRC screening at least once between 2014 and 2023. Second, we excluded those with a history of CRC in the Danish Cancer Registry^30^ or inflammatory bowel disease in the Danish National Patient Register^27^ prior to their first invitation. Finally, to retain age and sex balance between the 2 cohorts, we excluded people in the reference cohort who were matched to the excluded people with ID.

### CRC Screening

We obtained information on CRC screening from the Danish Colorectal Cancer Screening Database. The database contains information on all screening procedures and results, including invitations, participation, FIT results, and further diagnostic examination.^26^

We obtained data on overall participation (participation after all, some, or no invitations between 2014 and 2023). All other outcomes were anchored to the first screening to which the individuals were invited. Screening participation was defined as returning a stool sample within 90 days after the first invitation. Among those who participated after the first invitation (no time restriction), we retrieved information on FIT results defined as positive, negative, or nonanalyzable. We also obtained information on further diagnostic examination among those with a positive FIT result after first invitation, defined as undergoing colonoscopy, flexible sigmoidoscopy, or CT colonography within 60 days after positive FIT.^12^ Finally, we analyzed information on completion of colonoscopy (complete or incomplete). See eTable 2 in Supplement 1 for overview and definitions of the outcomes.

### Covariates

Age, sex, country of origin, somatic and psychiatric comorbidity were assumed to be potential confounders. We obtained information on age, sex and country of origin from the Danish Civil Registration System.^29^ Somatic comorbidity was defined by the Charlson Comorbidity Index^31^ on the basis of information on diagnoses from the National Patient Register^27^ registered up to 10 years prior to the first screening invitation. Charlson Comorbidity Index scores were categorized as 0, 1 to 2, and 3 or greater. Information about severe psychiatric comorbidity was also obtained from the National Patient Register,^27^ and defined as any contact to a psychiatric hospital department for other psychiatric diagnoses than ID (*ICD-10* codes F00-F69 and F80-F99) within the last 10 years prior to the first invitation.

### Statistical Analysis

We calculated proportions for all outcomes: returning stool sample within 90 days, FIT results, diagnostic examination within 60 days after positive FIT, and colonoscopy completeness. We estimated cumulative incidence (risk) differences and incidence ratios between people with and without ID for the outcomes of (1) returning a stool sample within 90 days after the first invitation, and (2) undergoing diagnostic examination within 60 days after positive FIT result. For these analyses we applied the pseudo-observations method, a time-to-event approach that enables direct regression modeling of the cumulative incidence function under competing risks and with right-censored data. This approach enables estimation of absolute and relative measures of association at specific time points. It involves 2 steps. First, pseudo-values for each individual were derived from the Aalen-Johansen estimator, representing event status at a specific time point without censoring. Second, a generalized linear model using the pseudo-values as the outcome was fitted, with a logistic or identity link to estimate risk ratios or risk differences, respectively.^32^ Death was included in the models as a competing event, even though this was not considered a major issue, as follow-up was short (90 and 60 days).

Multinominal logistic regression models were applied for the outcomes (1) FIT result (nonanalyzable vs positive, or negative result, and positive result vs negative result), and (2) completeness of colonoscopy after positive FIT result (incomplete vs complete). For all the outcomes, we conducted 2 sets of analyses. First, we compared the entire ID cohort with the reference cohort. Second, we examined ID severity as an independent variable to compare the specific severity groups with the reference cohort. We applied 2 models: one sex-adjusted and age-adjusted, and one further adjusted for country of origin and somatic and psychiatric comorbidities. Data were analyzed with Stata statistical software version 19 (StataCorp). Statistical significance was defined as a 95% CI that did not include the null value of 0 for adjusted cumulative incidence difference or 1 for incidence ratios and odds ratios.

## Results

The study population comprised 17 117 people with ID (median [IQR] age, 55.2 [50.2-63.2] years; 8445 female [49.3%]) and 149 162 people without ID (median [IQR] age, 54.8 [50.2-62.5] years; 75 324 female [50.5%]), all with no history of CRC or inflammatory bowel disease and invited for at least 1 CRC screening between 2014 and 2023 (see eFigure 1 in Supplement 1 for flowchart of inclusion). Information on ID severity was available for half of the ID population, with the majority having mild ID (Table 1).

**Table 1.** Characteristics of Study Cohort

Characteristic	Participants, No. (%)	
	People with ID (n = 17 117)	People without ID (n = 149 162)
Sex		
Male	8672 (50.7)	73 838 (49.5)
Female	8445 (49.3)	75 324 (50.5)
Age group, y		
49-50	6724 (39.3)	59 367 (39.8)
51-55	2291 (13.4)	20 957 (14.0)
56-60	2732 (16.0)	24 255 (16.3)
61-65	2265 (13.2)	19 654 (13.2)
66-70	1836 (10.7)	15 179 (10.2)
71-75	1269 (7.4)	9750 (6.6)
Birth cohorts		
1940-1949	2985 (17.4)	24 001 (16.1)
1950-1959	4872 (28.5)	42 534 (28.5)
1960-1969	6666 (38.9)	60 003 (40.2)
1970-1973	2594 (15.2)	22 624 (15.2)
Immigration status and country of origin^a^		
Danish origin	16 034 (93.7)	134 401 (90.1)
Immigrants or descendants from Western countries	177 (1.0)	5497 (3.7)
Immigrants or descendants from non-Western countries	906 (5.3)	9264 (6.2)
Somatic comorbidity^b^		
0	11 473 (67.0)	120 471 (80.8)
1-2	4453 (26.0)	23 992 (16.1)
≥3	1191 (7.0)	4699 (3.2)
Psychiatric comorbidity^c^		
No	12 002 (70.1)	140 591 (94.3)
Yes	5115 (29.9)	8571 (5.7)
ID severity		
Mild	5293 (30.9)	NA
Moderate	1974 (11.5)	NA
Severe	1113 (6.5)	NA
Profound	488 (2.9)	NA
Unknown	8249 (48.2)	NA

Abbreviations: ID, intellectual disability; NA, not applicable.

^a^ Western countries consist of all 28 European Union countries, as well as Andorra, Iceland, Liechtenstein, Monaco, Norway, San Marino, Switzerland, Vatican City, Canada, the US, Australia, and New Zealand. Non-Western countries consist of all other countries.

^b^ Somatic comorbidity is defined by the Charlson Comorbidity Index according to information on hospital-based diagnoses registered up to 10 years prior to the first screening invitation.

^c^ Psychiatric comorbidity is defined as any contact to a psychiatric hospital department for other psychiatric diagnoses than ID (*International Statistical Classification of Diseases and Related Health Problems, Tenth Revision* codes F00-F69 and F80-F99) within the last 10 years prior to the first invitation.

During the entire study period (2014 to 2023), 3366 people with ID (19.7%) participated in all screenings to which they were invited, whereas 9293 (54.3%) never participated. Among people without ID, 69 475 (46.6%) participated in all screenings, and 43 669 (29.3%) never participated (eTables 3 and 4 in Supplement 1).

In the following sections, all the results refer to the first screening to which the individuals were invited. See eFigure 2 in Supplement 1 for an overview of frequency of participation in screening, further diagnostic evaluation, and analyzability and completeness of these. Detailed results appear in Table 2, Table 3, Table 4, and Table 5. Only fully adjusted estimates are shown. Differences and ratios diminished slightly when adjusting for all confounders, but the patterns remained similar.

**Table 2.** Participation in Colorectal Screening Within 90 Days After First Invitation Among People With and Without ID^a^

Group	Participation in colorectal screening within 90 d after first invitation		
	Frequency, No. (%) of participants	Cumulative incidence difference, percentage points (95% CI)	Cumulative incidence ratio (95% CI)
No ID (n = 149 162)	83 709 (56.1)	0 [Reference]	1 [Reference]
ID (n = 17 117)	5170 (30.2)	−23.2 (−24.0 to −22.4)	0.56 (0.55 to 0.57)
ID severity			
Mild (n = 5293)	1453 (27.5)	−26.2 (−27.5 to −24.9)	0.51 (0.49 to 0.53)
Moderate (n = 1974)	572 (29.0)	−22.5 (−24.6 to −20.4)	0.56 (0.52 to 0.60)
Severe (n = 1113)	369 (33.2)	−19.5 (−22.4 to −16.7)	0.63 (0.58 to 0.68)
Profound (n = 488)	198 (40.6)	−13.4 (−17.8 to −9.1)	0.75 (0.67 to 0.83)
Unknown (n = 8249)	2578 (31.3)	−22.6 (−23.7 to −21.6)	0.57 (0.56 to 0.59)

Abbreviation: ID, intellectual disabilities.

^a^ Frequencies and proportions as well as cumulative incidence (risk) differences and ratios comparing screening participation among people with and without ID based on pseudo-observations and subsequent regression analyses adjusted for age, sex, immigration status and country of origin, and comorbidity.

**Table 3.** Result of First FIT Regardless of Time Between Invitation and Returning Stool Sample Among People With and Without ID^a^

Group	Result of first FIT, No. (%) of participants			OR (95% CI)	
	Positive	Negative	Nonanalyzable	Nonanalyzable vs positive or negative	Positive vs negative
No ID (n = 91 046)	5228 (5.7)	85 488 (93.9)	330 (0.4)	1 [Reference]	1 [Reference]
ID (n = 5894)	486 (8.2)	5303 (90.0)	105 (1.8)	4.49 (3.54-5.69)	1.35 (1.22-1.50)
ID severity					
Mild (n = 1651)	161 (9.8)	1445 (87.5)	45 (2.7)	6.93 (4.92-9.74)	1.50 (1.26-1.78)
Moderate to profound (n = 1370)	101 (7.4)	1261 (92.0)	8 (0.6)	1.50 (0.73-3.07)	1.16 (0.94-1.43)
Unknown (n = 2873)	224 (7.8)	2597 (90.4)	52 (1.8)	4.48 (3.31-6.05)	1.36 (1.18-1.57)

Abbreviations: FIT, fecal immunochemical test; ID, intellectual disabilities; OR, odds ratio.

^a^ Frequencies and proportions as well as ORs and 95% CIs from multinominal logistic regression models comparing people with and without ID and adjusted for age, sex, immigration status or country of origin, and comorbidity.

**Table 4.** Diagnostic Examination (Primarily Colonoscopy) Within 60 Days After Positive FIT Among People With and Without ID^a^

Group	Diagnostic examination within 60 d after positive FIT		
	Frequency, No. (%) of participants	Cumulative incidence difference, percentage points (95% CI)	Cumulative incidence ratio (95% CI)
No ID (n = 5237)	4724 (90.2)	0 [Reference]	1 [Reference]
ID (n = 492)	347 (70.5)	−17.9 (−22.1 to −13.7)	0.80 (0.75 to 0.85)
ID severity			
Mild (n = 165)	127 (77.0)	−11.5 (−18.2 to −4.7)	0.87 (0.79 to 0.95)
Moderate to profound (n = 100)	50 (50.0)	−38.5 (−48.5 to −28.5)	0.56 (0.46 to 0.69)
Unknown (n = 227)	170 (74.9)	−13.8 (−19.5 to −8.2)	0.85 (0.79 to 0.91)

Abbreviations: FIT, fecal immunochemical test; ID, intellectual disabilities.

^a^ Frequencies and proportions as well as cumulative incidence (risk) differences and ratios comparing screening diagnostic examination among people with and without ID based on pseudo-observations and subsequent regression analyses adjusted for age, sex, immigration status or country of origin, and comorbidity.

**Table 5.** Colonoscopy Completeness After Positive FIT Regardless of Time Between Positive FIT and Colonoscopy Among People With and Without ID^a^

Group	Colonoscopy completeness after positive FIT, No. (%) of participants			Incomplete bowel preparation vs complete colonoscopy, OR (95% CI)
	Complete	Incomplete, poor bowel preparation	Incomplete, other reason or missing information	
No ID (n = 4878)	4205 (86.2)	175 (3.6)	498 (10.2)	1 [Reference]
ID (n = 387)	278 (71.8)	43 (11.1)	66 (17.1)	2.07 (1.61-2.66)
ID severity				
Mild (n = 141)	105 (74.5)	14 (9.9)	22 (15.6)	1.68 (1.12-2.52)
Moderate to profound (n = 62)	37 (59.7)	8 (12.9)	17 (27.4)	3.62 (2.12-6.18)
Unknown (n = 184)	136 (73.9)	21 (11.4)	27 (14.7)	1.97 (1.39-2.79)

Abbreviations: FIT, fecal immunochemical test; ID, intellectual disabilities; OR, odds ratio.

^a^ Frequencies and proportions as well as ORs and 95% CIs from multinominal logistic regression models comparing people with and without ID and adjusted for age, sex, immigration status or country of origin, and comorbidity.

### Screening Participation

People with ID were less likely to participate in CRC screening after their first invitation compared with those without ID (Table 2). Overall, 5170 people with ID (30.2%) returned a stool sample within 90 days after their first invitation compared with 83 709 people (56.1%) without ID (adjusted cumulative incidence difference, −23.2 percentage points; 95% CI, −24.0 to −22.4 percentage points; cumulative incidence ratio, 0.56; 95% CI, 0.55 to 0.57). Participation increased with severity of ID, ranging from 1453 of 5293 individuals with mild ID (27.5%) to 198 of 488 individuals with profound ID (40.6%).

### Results of CRC Screening

Most stool samples were analyzable and negative (Table 3). However, the proportion of nonanalyzable stool samples was higher among people with ID (105 individuals [1.8%]) than those without ID (330 individuals [0.4%]) (adjusted odds ratio [OR], 4.49; 95% CI, 3.54 to 5.69). Nonanalyzable stool samples were especially frequent among people with mild ID (45 individuals [2.7%]). Additionally, people with ID were more likely to have a positive FIT result than those without (adjusted OR, 1.35; 95% CI, 1.22 to 1.50) (Table 3).

### Diagnostic Examination Among Those With Positive Screening

People with ID were less likely to undergo further diagnostic examination (mainly colonoscopy) within 60 days after a positive FIT result (Table 4). Overall, 347 people with ID (70.5%) underwent diagnostic examination compared with 4724 people (90.2%) without ID (adjusted cumulative incidence difference, −17.9 percentage points; 95% CI, −22.1 to −13.7 percentage points; cumulative incidence ratio, 0.80; 95% CI, 0.75 to 0.85). The proportion who underwent diagnostic examination decreased with increasing ID severity ranging from 127 of 165 individuals with mild ID (77.0%) to 50 of 100 individuals with moderate to profound ID (50.0%).

Colonoscopies were more likely to be incomplete among people with ID (109 individuals [28.2%]) compared with those without ID (673 individuals [13.8%]), and a larger proportion of these were explained by poor bowel preparation (adjusted OR for incomplete colonoscopy among people with ID vs those without ID, 2.07; 95% CI, 1.61 to 2.66) (Table 5). The largest proportion of incomplete colonoscopies was found among people with moderate to profound ID (25 individuals [40.3%]).

## Discussion

In this large nationwide cohort study, we found markedly lower participation in CRC screening and further diagnostic examination among people with ID compared with those without ID. The proportion of returned stool samples increased with the severity of ID, whereas the proportion receiving undergoing examination after a positive screening test result decreased. The proportions of nonanalyzable stool samples and incomplete colonoscopies were higher among people with ID compared with those without ID.

The markedly low screening participation among people with ID found in this study is in line with previous studies.^14,15,16,17,18,19,20,21,22^ The present study contributes to the existing knowledge with unique results on screening participation and completion across groups with different levels of ID severity, and on information from the entire screening pathway from invitation to further diagnostic examination following a positive FIT result.

Only 2 previous studies^14,18^ have examined screening participation according to ID severity. In line with the results from the present study, they showed that people with mild ID participate less in FITs than those with more severe levels of ID. To our knowledge, no studies have investigated the reasons for this. A possible contributing factor is that people with more severe levels of ID are dependent on support from professional carers who might assist them in collecting the stool sample. Many people with mild ID live by themselves and may not have any support to collect and return the sample. This may also explain why we found a larger proportion of nonanalyzable stool samples from people with mild ID than from the other groups. The instruction letter is not targeted at people with ID and is most likely challenging for them to comply with.

An interesting finding was the inverse association between ID severity and undergoing diagnostic examination after a positive FIT result. At this step of the screening pathway, the proportion who were examined decreased with increasing severity of ID. Thus, only half of those with moderate to profound ID who had a positive FIT result underwent further diagnostic examination. This raises concerns regarding inadequate informed decision-making. If this is an indication of lack of knowledge on the implications of a positive FIT result when the stool sample is taken, there is an urgent need to provide better information to people with ID, their families, and professional caregivers regarding the implications of screening. Guidance from a health care professional may be needed already at this stage of the screening pathway. If further diagnostic examination following a positive FIT result is hindered or treatment options after a cancer diagnosis are reduced due to the general health status of the individual or the severity of the ID, it is not ethically reasonable to initiate the screening process by sending in a stool sample. There is an immediate need for studies that examine what people with ID and their caregivers need in order to make an informed decision on whether participation is relevant for the individual.

A few studies have suggested different reasons for this markedly lower cancer screening participation among people with ID. A recently published systematic review^33^ found that barriers for screening participation in this group are perceptions of fear, distress, and embarrassment; unpreparedness for screening; negative interactions with health care professionals; a lack of knowledge about cancer screening; mobility issues; and a lack of ability to provide consent and communicate verbally. As seen from the present study, even though some individuals with ID overcome these barriers and participate in CRC screening, they are still more likely to experience challenges with stool sample collection and colonoscopy completion (eg, due to poor bowel preparation). Taken together, it seems that the screening program is not sufficiently tailored to people with ID. There is an urgent need to discuss and consider targeted approaches for cancer screening in this group, and as our results show that issues with different steps of the screening pathway vary according to level of severity of ID, differentiated approaches might be needed for different groups of people with ID.

### Strengths and Limitations

To our knowledge, this is the first study that investigates participation in the Danish CRC screening program for people with ID. The screening program is tax financed, and, thus, access to screening is independent of income and health insurance. Owing to the population-based design, the results can be generalized to countries with similar health systems and screening programs. We have established a population-based cohort of people with ID from a range of registers, which minimizes the risk of selection bias. Furthermore, we have utilized high-quality detailed data from a clinical database on all screening procedures and results, thus minimizing the risk of information bias.

However, there are a few limitations to be mentioned. People with ID are not systematically registered in Denmark, an although we used a range of high-quality data sources to identify them, people with mild ID may be underrepresented, since they do not reside at institutions, and a few might not have had any hospital contacts. In theory, some of those people who are not captured in the ID cohort (but have ID) could have ended up in the control cohort, but the effect of this is neglectable as ID prevalence in the general population is low. Another limitation is the missing information on ID severity for half of the population. This included people who were registered with *ICD-10* codes F78 (other mental retardation) or F79 (mental retardation without specification) or a diagnosis without specification of severity (eg, Down syndrome or cerebral palsy), and those who were identified through institutions (no information on diagnosis). In addition, although examining the influence of physical disability or social support on screening participation would have been relevant, this information was not available in the registers.

## Conclusions

In this nationwide, register-based cohort study of people with and without ID, those with ID were markedly less likely to participate in CRC screening and, when they did, faced more challenges with stool sample collection and colonoscopy completion. The patterns in screening participation and completion varied by ID severity. The disparities in screening may contribute to later-stage diagnosis, underscoring the need for tailored strategies to ensure equitable access to CRC screening for this vulnerable group. A cornerstone in such strategies should include sufficient support for informed decision-making regarding screening participation.

## Notes

Supplement 1. eFigure 1. Flowchart of inclusion eFigure 2. Flowchart of participation in and results of screening and diagnostic examination after first invitation to screening eTable 1. Diagnoses used to identify persons with intellectual disabilities (ID) eTable 2. Overview and definition of outcomes eTable 3. Overall screening participation (2014-2023) among people with and without intellectual disability eTable 4. Overall screening participation (2014-2023) among people with and without intellectual disability that received at least two invitations

Supplement 2. Data Sharing Statement
