# Supplementary material for: Colorectal Cancer Screening Among People With Intellectual Disabilities
Source: JAMA Netw Open. 2026 Jan 30;9(1):e2557013. doi: 10.1001/jamanetworkopen.2025.57013 (PMC12859718; doi:10.1001/jamanetworkopen.2025.57013)
Supplement: Supplement 1. — eFigure 1. Flowchart of inclusion eFigure 2. Flowchart of participation in and results of screening and diagnostic examination after first invitation to screening eTable 1. Diagnoses used to identify persons with intellectual disabilities (ID) eTable 2. Overview and definition of outcomes eTable 3. Overall screening participation (2014-2023) among people with and without intellectual disability eTable 4. Overall screening participation (2014-2023) among people with and without intellectual disability that received at least two invitations [file jamanetwopen-e2557013-s001.pdf]

Supplemental Online Content

Horsbøl TA, Michelsen SI, Sørensen TT, et al. Colorectal cancer screening among people with intellectual disabilities. *JAMA Netw Open*. 2026;9(1):e2557013.
doi:10.1001/jamanetworkopen.2025.57013

eFigure 1. Flowchart of inclusion

eFigure 2. Flowchart of participation in and results of screening and diagnostic examination after first invitation to screening

eTable 1. Diagnoses used to identify persons with intellectual disabilities (ID)

eTable 2. Overview and definition of outcomes

eTable 3. Overall screening participation (2014-2023) among people with and without intellectual disability

eTable 4. Overall screening participation (2014-2023) among people with and without intellectual disability that received at least two invitations

This supplemental material has been provided by the authors to give readers additional information about their work.

© 2026 Horsbøl TA et al. *JAMA Network Open*.

eFigure 1. Flowchart of inclusion.

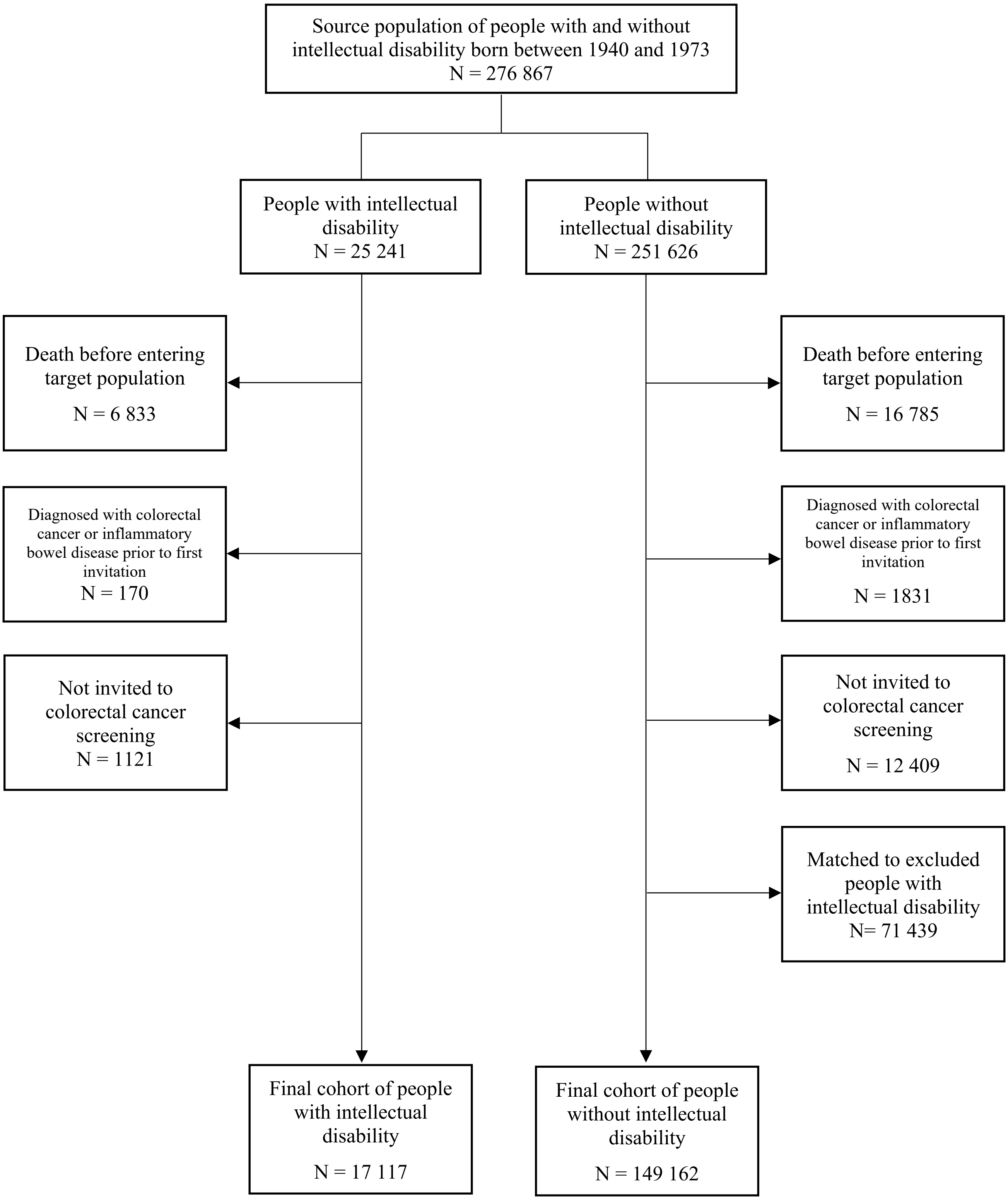

© 2026 Horsbøl TA et al. *JAMA Network Open*.

eFigure 2. Flowchart of participation in and results of screening and diagnostic examination after first invitation to screening.

Horizontal arrows follow the entire study population through screening and further diagnostic examination, while vertical arrows lead to analyses with time restrictions on participation, which also is described in detail in Tables 2-5.

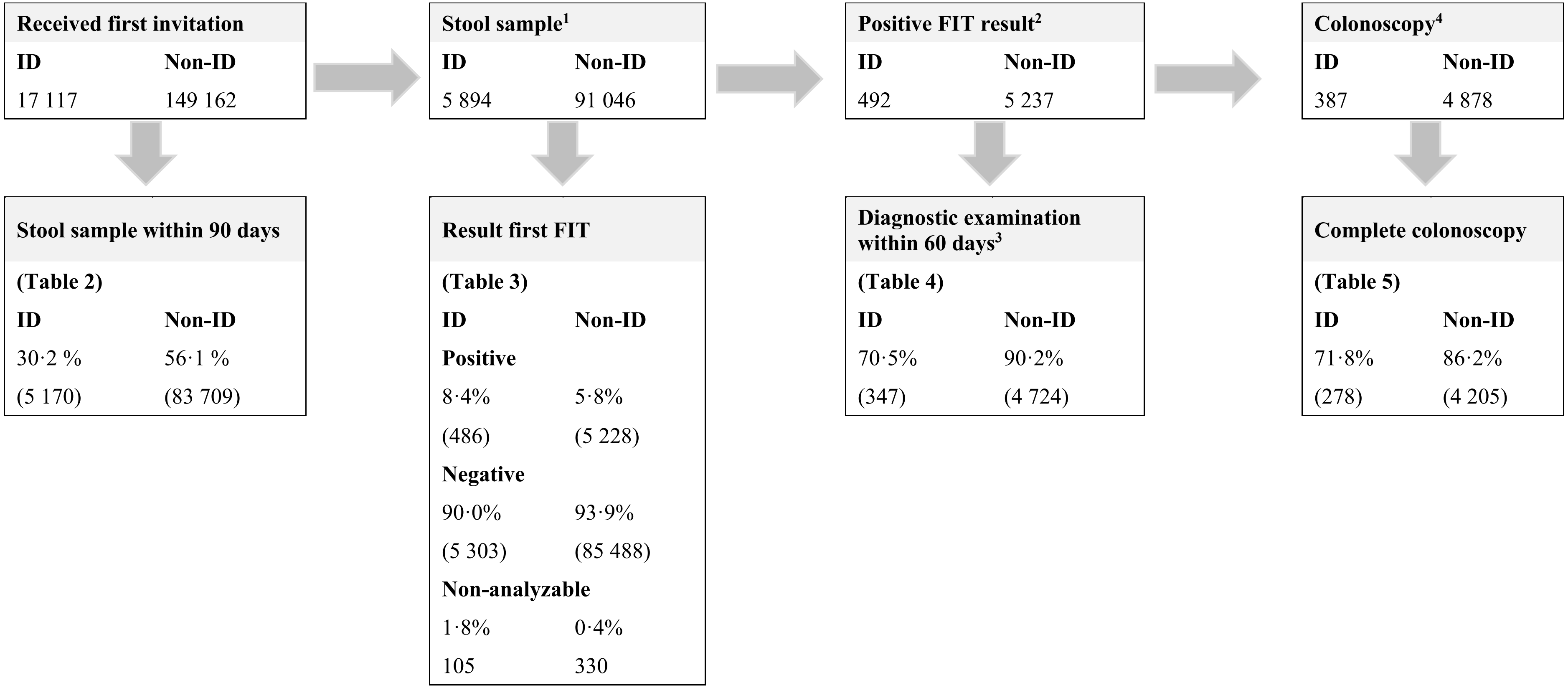

ID: Intellectual disability
[1]Regardless of time after invitation; [2]Include results of extra stool samples submitted after non-analyzable samples; [3]Primarily colonoscopy but also flexible sigmoidoscopy or CT colonography; [4]Regardless of time after positive FIT result.

© 2026 Horsbøl TA et al. *JAMA Network Open*.

eTable 1. Diagnoses used to identify persons with intellectual disabilities (ID).

Diagnosis	ICD-8	ICD-10
Mild ID	310-311	F70
Moderate ID	312	F71
Severe ID	313	F72
Profound ID	314	F73
Other ID and ID without further specification	315	F78, F79
Cerebral palsy and ID	-	G80[a]
Downs syndrome	759.3	Q90
Metabolic disorders likely to result in ID	271.2	E72.0E, E72.5A, E72.8E, E74.2B, E74.4B, E74.4C, E75.0, E75.1, E75.2D, E75.2E, E75.2G, E75.2H, E75.3, E75.4, E75.5A, E75.5B, E77, E79.1
Congenital malformation and chromosomal disorders likely to result in ID	759.6	Q85.1, Q93.5C, Q93.8A, Q99

[a] People with cerebral palsy were not identified by ICD-10 codes but through child neurologist medical record review.
Abbreviations: ID, intellectual disabilities

© 2026 Horsbøl TA et al. *JAMA Network Open*.

eTable 2. Overview and definitions of outcomes.

**All invitations**		
**Outcome**	**Definition**	**Population**
Overall participation	Participation after all, some, or no invitations between 2014 and 2023	People that were invited to screening at least once/twice between 2014 and 2023
**First invitation**		
**Outcome**	**Definition**	**Population**
Screening participation	Returning a stool sample within 90 days after the first invitation	People that were invited to screening at least once between 2014 and 2023
Screening test result	Positive, negative, or non-analyzable FIT	People that returned a stool sample after first invitation
Further diagnostic examination	Colonoscopy, flexible sigmoidoscopy, or CT colonography within 60 days after positive result	People that had a positive FIT result after first screening
Completion of colonoscopy	Complete, incomplete (poor bowel preparation), incomplete (other reason or missing info)	People that attended colonoscopy

Abbreviations: FIT, faecal immunochemical test

eTable 3. Overall screening participation (2014-2023) among people with and without intellectual disabilities.

	**Always No. (%)**	**Sometimes No. (%)**	**Never No. (%)**
**No ID** (n=149 162)	69 475 (46.6)	36 018 (24.1)	43 669 (29.3)
**ID** (n=17 117)	3 366 (19.7)	4 458 (26.0)	9 293 (54.3)
**ID Severity**			
Mild (n=5 293)	957 (18.1)	1 237 (23.4)	3,099 (58.5)
Moderate (n=1 974)	306 (15.5)	621 (31.4)	1 047 (53.0)
Severe (n=1 113)	236 (21.2)	383 (34.4)	494 (44.4)
Profound (n=488)	129 (26.4)	187 (38.3)	172 (35.2)
Unknown (n=8 249)	1 738 (21.1)	2 030 24.6)	4 481 (54.3)

Abbreviations: ID, intellectual disabilities

© 2026 Horsbøl TA et al. *JAMA Network Open*.

eTable 4. Overall screening participation (2014-2023) among people with and without intellectual disabilities that received at least two invitations.

	**Always No. (%)**	**Sometimes No. (%)**	**Never No. (%)**
**No ID** (n=124 884)	56 414 (45.2)	36 018 (28.8)	32 452 (26.0)
**ID** (n=12 972)	2 140 (16.5)	4 458 (34.4)	6 374 (49.1)
**ID Severity**			
Mild (n=4 065)	640 (15.7)	1 237 (30.4)	2 188 (53.8)
Moderate (n=1 477)	173 (11.7)	621 (42.0)	683 (46.2)
Severe (n=795)	122 (15.3)	383 (48.2)	290 (36.5)
Profound (n=352)	72 (20.5)	187 (53.1)	93 (26.4)
Unknown (n=6 283)	1 133 (18.0)	2 030 (32.3)	3 120 (49.7)

Abreviations: ID, intellectual disabilities

© 2026 Horsbøl TA et al. *JAMA Network Open*.
