# Supplementary material for: Colorectal Cancer Screening Among People With Intellectual Disabilities
Source: JAMA Netw Open. 2026 Jan 30;9(1):e2557013. doi: 10.1001/jamanetworkopen.2025.57013 (PMC12859718; doi:10.1001/jamanetworkopen.2025.57013)
Supplement: Supplement 2. — Data Sharing Statement [file jamanetwopen-e2557013-s002.pdf]

Data Sharing Statement

Horsbøl. Colorectal Cancer Screening Among People With Intellectual Disabilities. *JAMA Netw Open*. Published January 29, 2026. doi:10.1001/jamanetworkopen.2025.57013

Data
Data available: No

Additional Information
Explanation for why data not available: The data underlying this study are available only through Statistics Denmark and cannot be shared publicly due to Danish data-protection regulations. Access to the data requires secure-server permissions granted by Statistics Denmark, and the authors are not permitted to distribute the data directly; interested researchers may apply for access through Statistics Denmark in accordance with their institutional requirements.
